# Constrained Active Learning for Anchor Link Prediction Across Multiple Heterogeneous Social Networks

**DOI:** 10.3390/s17081786

**Published:** 2017-08-03

**Authors:** Junxing Zhu, Jiawei Zhang, Quanyuan Wu, Yan Jia, Bin Zhou, Xiaokai Wei, Philip S. Yu

**Affiliations:** 1 College of Computer, National University of Defense Technology, Changsha 410073, China; qywu@nudt.edu.cn (Q.W.); jiayanjy@vip.sina.com (Y.J.); binzhou@nudt.edu.cn (B.Z.); 2 Department of Computer Science, Florida State University, Tallahassee, FL 32306-4530, USA; jwzhanggy@gmail.com; 3 Department of Computer Science, University of Illinois at Chicago, Chicago, IL 60607, USA; weixiaokai@gmail.com (X.W.); psyu@cs.uic.edu (P.S.Y.)

**Keywords:** multiple heterogeneous social networks, anchor link prediction, Constrained Active Learning

## Abstract

Nowadays, people are usually involved in multiple heterogeneous social networks simultaneously. Discovering the anchor links between the accounts owned by the same users across different social networks is crucial for many important inter-network applications, e.g., cross-network link transfer and cross-network recommendation. Many different supervised models have been proposed to predict anchor links so far, but they are effective only when the labeled anchor links are abundant. However, in real scenarios, such a requirement can hardly be met and most anchor links are unlabeled, since manually labeling the inter-network anchor links is quite costly and tedious. To overcome such a problem and utilize the numerous unlabeled anchor links in model building, in this paper, we introduce the active learning based anchor link prediction problem. Different from the traditional active learning problems, due to the *one-to-one constraint* on anchor links, if an unlabeled anchor link a=(u,v) is identified as positive (i.e., existing), all the other unlabeled anchor links incident to account *u* or account *v* will be negative (i.e., non-existing) automatically. Viewed in such a perspective, asking for the labels of potential positive anchor links in the unlabeled set will be rewarding in the active anchor link prediction problem. Various novel anchor link information gain measures are defined in this paper, based on which several constraint active anchor link prediction methods are introduced. Extensive experiments have been done on real-world social network datasets to compare the performance of these methods with state-of-art anchor link prediction methods. The experimental results show that the proposed *Mean-entropy-based Constrained Active Learning (MC)* method can outperform other methods with significant advantages.

## 1. Introduction

Online social networks have become more and more popular in recent years, and are often represented as heterogeneous information networks containing abundant information about: who, where, when and what [1]. Different social networks may have different functionalities, so it is natural for individuals to use multiple social networks for different purposes at the same time [2,3]. For example, an individual may use Facebook to share funny posts with his/her friends, use Twitter to follow the latest news and events, and use Foursquare to search for the places of interest in his/her surrounding area. However, the accounts owned by the same user in different social sites are mostly isolated without any correspondence connections to each other.

Linking the accounts of the same person across different social networks is of great value for many concrete real-world inter-network applications [4,5,6,7]. For example, after aligning Facebook and Twitter, we can recommend new friends or new topics to a new Twitter user according to the social relationship or personal interest information from his/her existing Facebook account. Meanwhile, if we can effectively align Twitter and Foursquare, we can also recommend new places to a new Foursquare user by analyzing the location check-in records about him/her in Twitter. The correspondence relationships connecting common users’ accounts across different social networks are called the “anchor links” [4].

In order to predict anchor links between multiple social networks, many different supervised methods have been proposed so far. However, these existing methods can achieve good performance only when sufficient labeled anchor links can be collected to train the models [1,8,9,10,11,12]. In these supervised anchor link prediction methods, the anchor link prediction is modeled as a classification problem, where the existing and non-existing anchor links are labeled as positive and negative instances respectively. In the scenarios when users’ personal profile information (e.g., email, phone number and address) is available on the social networks, by directly searching and matching these information, manually labeling the anchor links as the training set will not be a problem. However, in most cases, social network data available for research is usually anonymized for privacy concerns [13], where users’ profile information is either removed or replaced with meaningless unique identifiers. Therefore, the majority of the anchor links between social networks are actually unknown and can be extremely time-consuming for manually labeling (e.g., manually ask the user of a given account u1 that whether an account u2 in another network also belongs to him/her, and if he/she says “yes”, then label the link between u1 and u2 as a positive anchor link) [2]. With such limited labeled anchor links, none of the existing classification-based methods (training of which requires lots of labeled instances) can perform well. One way to solve this challenging problem is to exploit the active learning technique to utilize a reasonable-sized labeled anchor links together with the numerous unlabeled anchor links to improve the model building.

In contrast to using randomly selected labeled data to induce a model, active learning gives the learners the flexibility to select which instances to be labeled and added to the training set [14]. In this way, the active learner aims to achieve high accuracy using as few labeled instances as possible, and thereby minimizing the cost of obtaining labeled data [15]. However, many existing active learning methods [16,17,18,19] just focus on data that is assumed to be independent and identically distributed, where the objects either do not have explicit relationships with one another, or the relationships have been ignored [20]. Some link-based active learning methods have been proposed to deal with intra-network links [20,21,22]. However, the problems explored by them are very different from the active anchor link prediction problem: (1) anchor links are the links that connect different networks. Compared with the intra-network links, they are often very hard to collect and contain the information between multi-source networks; (2) most of the intra-network links have no cardinality constraint, but anchor links normally follow the *one-to-one constraint* [1], i.e., each user can have at most one account in each social network. (The case that users have multiple accounts in one network is a different problem [23]. However, in the problem of anchor link prediction, it can be resolved with method introduced in [24], where these duplicated accounts can be aggregated in advance to form one unique virtual account and the constraint on anchor links connecting these virtual accounts will still be “one-to-one”.)

As a result, to apply active learning on anchor link prediction, there are several challenges to be solved:

- *one-to-one constraint on anchor links*: anchor links have an inherent *one-to-one constraint* [1], which has never been considered in traditional active learning methods at all. Via the *one-to-one constraint*, when identifying one positive anchor link, a group of negative anchor links incident to its nodes can be discovered from the networks. Viewed in such a perspective, identifying positive anchor links and using the *one-to-one constraint* to infer their related negative links will be very important for the active anchor link prediction problem.
- *sparsity of anchor links*: unlike other kinds of social network link, due to the *one-to-one constraint*, the positive anchor links between two given networks are extremely sparse, and only account for a small proportion among all the potential inter-network user pairs. As a result, when collecting the training set, acquiring enough positive anchor links under a limited cost is very challenging.
- *heterogeneity of social networks*: anchor links in online social networks can be associated with heterogeneous information, like various types of attributes and complex connections [1]. How to properly apply such heterogeneous information to the active learning for anchor link prediction is quite different from traditional active learning and link prediction problems.

This paper is the first work to introduce several active learning methods to tackle the anchor link prediction issues. In this paper, we propose our *Constrained Active Learning* methods. Different from the existing active learning methods, when identifying one positive anchor link, our methods can discover a group of negative anchor links that incident to its nodes via the *one-to-one constraint*, thus the challenge of *one-to-one constraint on anchor links* is solved. Besides, several mechanisms have been designed to ensure that enough informative positive anchor links can be queried by our methods, in this way to overcome the bad effects caused by the challenge of *sparsity of anchor links*. Meanwhile, we choose *MNA* [1], which is a state-of-art supervised method based on heterogeneous features, as the basic anchor link prediction method, so that to enable our approaches to deal with the challenge of *heterogeneity of social networks* very well.

The rest of the paper is organized as follows: We firstly make a simple introduction to the related works of our study in Section 2. Secondly, we formulate the problem in Section 3. In Section 4, we introduce the basic anchor link prediction method. Then we discuss some classical active learning methods, and propose our *Constrained Active Learning* methods. In Section 5, we run extensive experiments on real-world heterogeneous social networks, and discuss the experiment results. Finally, we conclude this paper.

## 2. Related Works

Social network link prediction problems have been explored for several years [25,26], and many different works on supervised social network link prediction have been studied [27,28,29]. Among them, Hasan et al. [27] are the first to study social link prediction as a supervised problem.

Different from traditional social network link prediction problems, anchor link prediction, focuses on predicting the existing anchor links between multiple networks. In addition, in recent years, many different supervised methods have been proposed to solve the anchor link prediction problems [1,7,8,9,10,11,12,30]. Among them, Vosecky et al. [12] propose a method to connect users between Facebook and StudiVZ baseed on web profile matching. Zafarani and Liu [9] first introduce a methodology for connecting identities across social networks based on multiple behavioral patterns of username creation. Liu et al. [11] propose a framework to connect user accounts across heterogeneous social media platforms by using multiple user features. Kong et al. [1] extract heterogeneous features from multiple heterogeneous networks for anchor link prediction, including user’s social, spatial, temporal and text information, and formulate the inference problem for anchor links as a stable matching problem. Goga et al. [30] conduct a systematic and detailed investigation of the reliably of matching user profiles across real-world online social networks, and propose a matching scheme that is able to mitigate impersonation attacks and reduce the number of false matches. In addition, Zhang et al. [7] develop a general cross-network user alignment model which can support the integration of a number of networks.

Since anchor links connect user accounts across two different network sources, and follow the *one-to-one constraint*, the information of two different networks can be directly transferred via these links. As a result, how to apply anchor links to cross-network applications becomes a new problem, and is explored by several works recently, for example: Zhang et al. [4,31] explore the ways of using anchor links to integrate the relation information from multiple networks to conduct social link prediction. Pan et al. [32] propose a matrix factorization method to transfer one network’s user latent factors to help the recommendation task in the other network to achieve better performances. Yan et al. [33,34] transfer users’ rich social and content information in Twitter network to help recommend the videos in YouTube.

Active learning, which sometimes is also called “query learning” or “optimal experimental design” in the statistics literature, has been researched by many works [15], and used in many applications. For example, image classification [35,36], biomedicine [37], system monitoring [38,39,40]. Most of the existing active learning methods [16,17,18,19] focus on data that is assumed to be independent and identically distributed, where the objects either do not have explicit relationships with one another, or the relationships have been ignored. Different from these methods, Bilgic et al. [20,21] utilize the relationships between the intra-network objects, and propose their link-based active learning theories. Xu et al. [22] apply the active learning to the area of preference learning by taking the information of pairwise judgments into consideration. Isele et al. [41] use active learning to help generate expressive linkage rules for entities, so that to solve the data integration problem. Xiong et al. [42] study the active learning problem of selecting pairwise must-link and cannot-link constraints for semi-supervised clustering. However, how to properly apply active learning to help predict the anchor links, which follow the *one-to-one constraint* and are not identically distributed, is still a new problem that remains to be explored.

## 3. Problem Formulation

Suppose there are a source network $G^{s}=\left(V^{s},L^{s}\right)$ and a target network $G^{t}=\left(V^{t},L^{t}\right)$, which are both heterogeneous social networks. The set of nodes in $G^{s}$ contains 4 kinds of nodes, and can be represented as $V^{s}=U^{s}\cup C\cup T\cup W$. $U^{s}=\left\{u_{1}^{s},u_{2}^{s},\cdots ,u_{N}^{s}\right\}$ is the set of user accounts in $G^{s}$. $C=\{c_{1},c_{2},\cdots ,c_{\left|C\right|}\}$ is the set of locations. $T=\{t_{1},t_{2},\cdots ,t_{\left|T\right|}\}$ represents a set of time slots that users have published posts at. $W=\{w_{1},w_{2},\ldots ,w_{\left|W\right|}\}$ is the set of words people have used in their posts. $L^{s}\subset V^{s}\times V^{s}$ is the intra-network links of different types in $G^{s}$. We define the target network $G^{t}$ in the similar way. $U^{t}$ denotes the set of user accounts in $G^{t}$. Without loss of generality, we assume $G^{s}$ and $G^{t}$ share the same sets of locations C, time slots T and words W.

**Supervised Anchor Link Prediction Problem**: Given $G^{s}$ and $G^{t}$, this problem aims at using the prediction model to infer the existing anchor links which connect users across these two networks. And in this paper, we define the set of anchor links between $G^{s}$ and $G^{t}$ as $A\;=\{a\left(u_{i}^{s},u_{j}^{t}\right)|u_{i}^{s}\;\in \;U^{s},u_{j}^{t}\;\in \;U^{t}\}$, where $a(u_{i}^{s},u_{j}^{t})$ represents the anchor link between two user accounts $u_{i}^{s}$ and $u_{j}^{t}$. If we are sure that $a(u_{i}^{s},u_{j}^{t})$ is an existing anchor link, we label it as “positive” and set its value as $a(u_{i}^{s},u_{j}^{t})=1$. However, if it is a non-existing anchor link, we label it as “negative” and set the its value as $a(u_{i}^{s},u_{j}^{t})=0$. Since anchor links are one-to-one relationships between user accounts in $U^{s}$ and $U^{t}$, we can present this constraint as $\forall i,\forall j(\sum_{k}a\left(u_{i}^{s},u_{k}^{t}\right)\leq 1,\sum_{k}a\left(u_{k}^{s},u_{j}^{t}\right)\leq 1)$. Let $A_{l}$ and $A_{u}$ denote the labeled and unlabeled anchor link set in A respectively, and $A_{t}\subset A_{l}$ denote the training set which is used to train the prediction model. Thus the task of supervised anchor link prediction is to train a prediction model from $A_{t}$ and use it to predict the value of a given unlabeled anchor link $a\left(u_{i}^{s},u_{j}^{t}\right)\in A_{u}$.

## 4. The *Constrained Active Learning* for Anchor Link Prediction

Active learning aims at minimizing the labeling cost of training set by letting the learner choose which examples to label. To anchor link prediction, the first thing that needs to be done by the active learner is to select a query pool P of unlabeled anchor links, whose links are randomly collected from the unlabeled anchor link set $A_{u}\subset A$. Thus we can present P as: $P=\{a\left(u_{i}^{s},u_{j}^{t}\right)|u_{i}^{s}\in U_{sub}^{s},u_{j}^{t}\in U_{sub}^{t},a\left(u_{i}^{s},u_{j}^{t}\right)\in A_{u}\}$, where $U_{sub}^{s}$ and $U_{sub}^{t}$ is the selected subset of $U^{s}$ and $U^{t}$ separately. Then the active learner will train an anchor link prediction model θ from the training set $A_{t}$. Finally, the active learner will begin a round of query, which is to identify the label(s) of the most valuable link(s) in P according to the values computed by conducting θ on P, and add the identified link(s) to $A_{t}$. The training process and query process will be repeated until the limit of query cost has been reached.

In this paper, instead of focusing on how to predict the existing anchor links, we focus on the active learning strategies. So in this section, we will just briefly introduce the basic anchor link prediction method, which can extract heterogeneous features from multiple heterogeneous networks and use them to predict anchor link. Then we will design several *Constrained Active Learning* methods for anchor link prediction, where these methods can be divided into the *Normal Constrained Active Learning* methods and the *Biased Constrained Active Learning* methods.

### 4.1. The Basic Anchor Link Prediction Method

A basic building block of our approach is *Multi-Network Anchoring (MNA)* which was proposed in [1]. It is a supervised method based on heterogeneous features and outperforms several famous classical methods on the anchor link prediction between Twitter and Foursquare. For the sake of completion, we now present the main idea of this approach in this subsection.

*MNA* firstly extracts 4 kinds of heterogeneous features from the source network $G^{s}$ and the target network $G^{t}$, including:

- *Multi-Network Social Features*: extracted by evaluating the similar social links of two user accounts from different social networks, to represent the social similarity between two user accounts.
- *Spatial Distribution Features*: extracted by comparing the location information of two user accounts in different ways, to represent people’s location similarities.
- *Temporal Distribution Features*: extracted by using different ways to compare the distribution of different users’ activities in the given time slots, to represent people’s temporal similarities.
- *Text Content Features*: extracted by evaluating the similarity of words used by two user accounts from different social networks, to represent the similarity of text contents posted by two user accounts.

Then *MNA* trains an *SVM* classifier on these extracted features, and uses it to predict the label of the unknown anchor links. Lastly, *MNA* uses a matching algorithm (referred to as *One-to-one Matching Algorithm*) to ensure the *one-to-one constraint* on anchor links, so that to make the predicted results more accurate and reasonable. The detailed description can be seen in [1].

### 4.2. The *Constrained Active Learning* Methods

**Figure 1:**
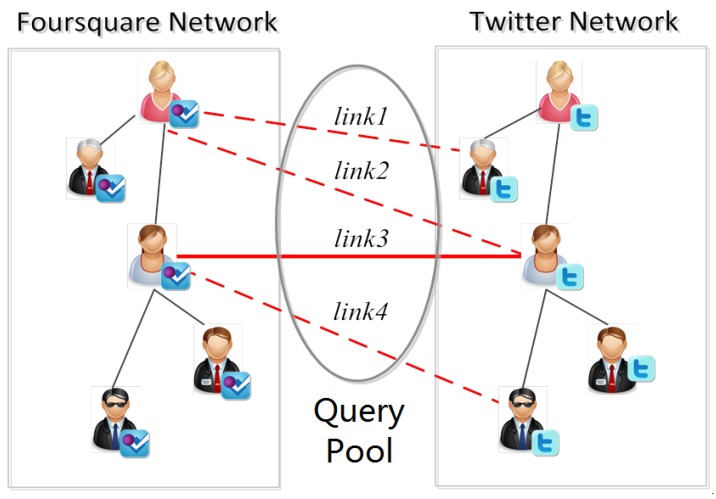
An example of active learning for anchor link prediction.

In each round of query process, traditional active learning methods usually just add the newly identified samples to the training set. However, via the *one-to-one constraint*, the *Constrained Active Learning* methods can infer the labels of some unlabeled links after identifying a positive anchor link, and thus the samples added to the training set can be more than the identified samples. As shown in Figure 1, there are 4 unlabeled anchor links in the query pool. After *link3* is identified as a positive anchor link in a round of query, a traditional active learner just adds *link3* to the positive training set. However, a constrained active learner will firstly infer that *link2* and *link4* are “negative” according to the *one-to-one constraint*, and then add *link3* to the positive training set and add *link2*, *link4* to the negative training set. In this way, the *Constrained Active Learning* methods can label more samples than the traditional active learning methods under the same query cost (where the query cost means the cost of identifying the selected unlabeled anchor link $a(u_{i}^{s},u_{j}^{t})$ in a round of query).

In this subsection, we will design several *Constrained Active Learning* methods, which can be divided into the *Normal Constrained Active Learning* methods and the *Biased Constrained Active Learning* methods. The former kind of methods treat the positive links and the negative links equally, while the later kind of methods pay more attention to the potential positive links when querying.

#### 4.2.1. The *Normal Constrained Active Learning* Methods

All active learning methods involve evaluating the informativeness of unlabeled instances. However, as mentioned in Section 1, due to the existing challenges, many query methods of active learning do not apply very well to anchor link prediction.

Among the existing query methods, the simplest and most commonly used query framework is *uncertainty sampling*, where the learner queries the instance about which it is least certain on how to label [15]. There are mainly 3 kinds of sampling strategies in *uncertainty sampling*, e.g., the *least confidence sampling* [14], the *margin sampling* [16] and the *entropy-based sampling* [14]. Compared with the former two sampling strategies, the *entropy-based sampling* generalizes more easily to complex structured instances. This is because by computing the entropies, we can compare the amount of information contained in different multi-structured samples in an uniform metric. So our *Constrained Active Learning* methods are based on the entropy theory, and aim to calculate the potential entropy $H_{p}\left(a\right)$ for each unlabeled link a∈P. Here we define $H_{p}\left(a\right)$ as the evaluated amount of information that the constraint active learner can gain by identifying the label of *a*.

Here, we use $R(a\left(u_{i}^{s},u_{j}^{t}\right))$ to represent the related link set of a given anchor link $a(u_{i}^{s},u_{j}^{t})$, and define it as the set of all the other anchor links in P that incident to node $u_{i}^{s}$ or $u_{j}^{t}$. For example, in Figure 1, the related linked set of *link3* is formed by *link2* and *link4*. Thus we can formulate $R(a\left(u_{i}^{s},u_{j}^{t}\right))$ as:

(1) $$R\left(a\left(u_{i}^{s},u_{j}^{t}\right)\right)=R_{s}\left(a\left(u_{i}^{s},u_{j}^{t}\right)\right)\cup R_{t}\left(a\left(u_{i}^{s},u_{j}^{t}\right)\right)$$

where $R_{s}\left(a\left(u_{i}^{s},u_{j}^{t}\right)\right)=\left\{a\left(u_{m}^{s},u_{j}^{t}\right)|\forall u_{m}^{s}\in U^{s},a\left(u_{m}^{s},u_{j}^{t}\right)\in P,m\neq i\right\}$ and $R_{t}\left(a\left(u_{i}^{s},u_{j}^{t}\right)\right)=\left\{a\left(u_{i}^{s},u_{n}^{t}\right)|\forall u_{n}^{t}\in U^{t},a\left(u_{i}^{s},u_{n}^{t}\right)\in P,n\neq j\right\}$.

The common main idea of the *Normal Constrained Active Learning* methods is to predict $H_{p}\left(a\left(u_{i}^{s},u_{j}^{t}\right)\right)$ for each unlabeled anchor link $a(u_{i}^{s},u_{j}^{t})\in P$, then identify the label of link $a_{h}\left(u_{i}^{s},u_{j}^{t}\right)$, which has the highest potential entropy among all links in P. If the label of $a_{h}\left(u_{i}^{s},u_{j}^{t}\right)$ is “negative”, then add $a_{h}\left(u_{i}^{s},u_{j}^{t}\right)$ to the training set $A_{t}$; otherwise, in addition to adding $a_{h}\left(u_{i}^{s},u_{j}^{t}\right)$ to $A_{t}$, the learner should find its related link set $R(a_{h}\left(u_{i}^{s},u_{j}^{t}\right))$ from P, then set the labels of all links in $R(a_{h}\left(u_{i}^{s},u_{j}^{t}\right))$ as “negative” and add this set to $A_{t}$.

By designing different ways to evaluate the potential entropy of each unlabeled anchor link $a(u_{i}^{s},u_{j}^{t})\in P$, we present two kinds of *Normal Constrained Active Learning* methods as follows:

- *The Basic-entropy-based Constrained Active Learning (BC)*: Using this method, the active learner calculates $H_{p}\left(a\right)$ of an unlabeled link *a* by its basic entropy $H_{B}\left(a\right)$. Here, the basic entropy can also be viewed as the amount of information contained in the link. To a given unlabeled anchor link *a*, the basic entropy of it is as follows:
  (2) $$H_{B}\left(a\right)=-\sum_{y=0}^{1}P_{\theta }\left(y|a\right)\log P_{\theta }\left(y|a\right)$$
  Here, $P_{\theta }\left(y|a\right)$ is the posterior probability of link *a*’s value to be *y* under the prediction model θ.
- *The Mean-entropy-based Constrained Active Learning (MC)*: different from *BC*, *MC* calculates $H_{p}\left(a\right)$ for an unlabeled anchor link *a* not only by its own entropy, but also by the mean entropy of all the links in *a*’s related link set R(a).

Now we present the details for *Mean-entropy-based Constrained Active Learning (MC)*:

In real scenarios, if the value of the newly identified link *a* is 0, the amount of information the learner can acquire is:

$$H_{a}\left(a^{-}\right)=H_{B}\left(a\right)$$

However, if the value of *a* is 1, the acquired amount of information will be:

$$H_{a}\left(a^{+}\right)=H_{B}\left(a\right)+\sum_{a_{r}}^{R(a)}H_{B}\left(a_{r}\right)$$

Since the learner will find the *a*’s related link set R(a), and label each link $a_{r}\in R\left(a\right)$ as “negative” according to the *one-to-one constraint*. As a result, the new information that will be finally acquired by the learner after identifying an unlabeled link *a* can be more than $H_{B}\left(a\right)$. Thus, to an unlabeled link *a*, one way to calculate $H_{a}\left(a\right)$ is to combine $H_{a}\left(a^{-}\right)$ and $H_{a}\left(a^{+}\right)$ by *a*’s probability to be 0 and 1, which is as follows:

(3) $$\begin{array}{cc} H_{a}\left(a\right) & =P_{M}\left(0|a\right)H_{a}\left(a^{-}\right)+P_{M}\left(1|a\right)H_{a}\left(a^{+}\right)\\ & =H_{B}\left(a\right)+P_{M}\left(1|a\right)\sum_{a_{r}}^{R(a)}H_{B}\left(a_{r}\right) \end{array}$$

where $P_{M}\left(y|a\right)$ is the probability of *a*’s value to be predicted as *y* by the trained *MNA* model *M*, thus we can make sure that $\sum_{y=0}^{1}P_{M}\left(y|a\right)=1$.

Although the *Platt scaling* can be used to evaluating the outputs of SVM into a probability distribution over classes, however, since the outputs of *MNA* are just predicted positive links without any of the classifier scores [1], the *Platt scaling* can not be used to compute $P_{M}\left(y|a\right)$. In order to compute $P_{M}\left(y|a\right)$, we should firstly select a labeled anchor link set $A_{v}$, and use it as the validation set to test the values of $P_{M}\left(0|a\right)$ and $P_{M}\left(1|a\right)$. Thus we formulate $A_{v}$ as follows:

$$A_{v}=\left\{a\left(u_{i}^{s},u_{j}^{t}\right)|a\left(u_{i}^{s},u_{j}^{t}\right)\in A_{sl},a\left(u_{i}^{s},u_{j}^{t}\right)\notin A_{t}\right\}$$

where $A_{sl}\subset A_{l}$, and $A_{l}$ is original labeled anchor link set which is collected before the active learning process. Each time, after training the classifier of *MNA* (To *MNA*, only its classifier need to be trained before we use it [1].) and before computing the entropy of the links in P, we should firstly pretend that we do not know the labels of all the links in $A_{v}$, and use the trained *MNA*’s model *M* to predict the labels for these links. Thus we can get two link sets $A_{v}^{+}$ and $A_{v}^{-}$, which consist of the predicted positive links and negative links separately. Supposing that N(A) is the number of links in a link set A, and $N^{+}\left(A\right)$ is the number of links in A whose real labels are “positive”, we can compute two probabilities $P_{YY}$ and $P_{NY}$ as follows:

(4) $$P_{YY}=\frac{N^{+}\left(A_{v}^{+}\right)}{N(A_{v}^{+})},P_{NY}=\frac{N^{+}\left(A_{v}^{-}\right)}{N(A_{v}^{-})}$$

Finally, in the querying process, if the predicted value of link a∈P is 1, set $P_{M}\left(1|a\right)=P_{YY}$, $P_{M}\left(0|a\right)=1-P_{YY}$; if the predicted value of link *a* is 0, set $P_{M}\left(1|a\right)=P_{NY},P_{M}\left(0|a\right)=1-P_{NY}$.

However, in real-world anchor link prediction problem, data samples are usually imbalanced, which means the negative anchor links can be much more than the positive anchor links. So it is important to make sure the identified positive links contain enough information in the query process. When we set $H_{p}\left(a\right)=H_{a}\left(a\right)$, for a positive link a∈P, if it has a large $P_{M}\left(1|a\right)$ and an informative related link set formed by many negative links, the value of $H_{a}\left(a\right)$ can be very large. In addition, thus *a* is likely to be identified in the query process, no matter whether it contains enough information. As a result, many positive links with little information in them but have informative related link sets are likely to be identified in the query process. This is contrary to our goal of making sure the identified positive links contain enough information. Our *MC* method overcomes this problem by modifying $H_{a}\left(a\right)$ to $H_{M}\left(a\right)$. $H_{M}\left(a\right)$ aims to calculate $H_{p}\left(a\right)$ for an unlabeled anchor link *a* according to its basic entropy and the mean entropy of the links in R(a). The formulation is as follows:

(5) $$H_{M}\left(a\right)=H_{B}\left(a\right)+P_{M}\left(1|a\right)\frac{\sum_{a_{r}}^{R(a)}H_{B}\left(a_{r}\right)}{N(R(a))}$$

where $\frac{\sum_{a_{r}}^{R(a)}H_{B}\left(a_{r}\right)}{N(R(a))}$ denotes the mean entropy of all the links in R(a). In addition, we can notice that for $H_{M}\left(a\right)$, the basic entropy of *a* is as important as the mean entropy of all the links in R(a). So $H_{M}\left(a\right)$ is more suitable for dealing with the data imbalance problem: (1) No matter how imbalance the data is, the active leaner can always pay attention to find the positive links which contain enough information. Because the value of $\frac{\sum_{a_{r}}^{R(a)}H_{B}\left(a_{r}\right)}{N(R(a))}$ doesn’t increase with the number of links in R(a), and thus for a large R(a), the computation of $\frac{\sum_{a_{r}}^{R(a)}H_{B}\left(a_{r}\right)}{N(R(a))}$ still cannot dominate the evaluation of the potential entropy of *a*. In addition, since basic entropy of *a* is as important as $\frac{\sum_{a_{r}}^{R(a)}H_{B}\left(a_{r}\right)}{N(R(a))}$, we can pay enough attention to identify the informative positive links; (2) No matter how imbalance the data is, the active learner can always ensure that for any identified positive link *a*, the negative links in R(a) are likely to be informative. Because the value of R(a) is evaluated by the mean entropy of all the links in R(a), and is not directly influenced by the imbalance degree of the data samples.

According to the definition of Equation (5), the validation set should contain sufficient labeled anchor links to make *MC* perform well. If the labeled anchor links in the validation set are not sufficient, the sample distribution of it can hardly represent the real world sample distribution, which is similar to the sample distribution of the query pool. As a result, the computed $P_{M}\left(1|a\right)$ can hardly adapt to the query pool, and thus when using *MC* to query samples in the query pool, the computed $H_{M}\left(a\right)$ values are not precise enough, which can result in the bad performances of *MC*.

The proposed framework of the *Normal Constrained Active Learning* is shown in Algorithm 1, if we set $H_{p}=H_{B}$, then the algorithm is *BC*; and if we set $H_{p}=H_{M}$, then the algorithm is *MC*.

**Algorithm 1** The framework of *Normal Constrained Active Learning***Input:** Two heterogeneous social networks: $G^{s}$ and $G^{t}$. Two sets of labeled anchor link: The training set $A_{t}$ and the validation set $A_{v}$. The query pool P. The max number of queries $n_{q}$. The potential entropy computation method $H_{p}$.**Output:** The new training set $A_{t}$ and the new query pool P1:Initialize n←02:For each $a(u_{i}^{s},u_{j}^{t})$ in $A_{t}$ and P, extract four types of features.3:**while** $n<n_{q}$ **do**4:    Train an *SVM* model θ on $A_{t}$ according to the training part in *MNA*;5:    For each unlabeled anchor link *a* in P, use θ to predict the probabilities of its value to be 0 and 1, which is presented as $P_{\theta }\left(0|a\right)$ and $P_{\theta }\left(1|a\right)$.6:    **if** $H_{p}$ is $H_{B}$ **then** for each link *a* in P, compute $H_{p}\left(a\right)$ by Equation (2)7:    **else**8:        Use θ as the trained classifier in *MNA*, and use *MNA* to predict the labels of all the links in $A_{v}$.9:        Compute the probabilities $P_{YY}$ and $P_{NY}$ by Equation (4).10:        For each *a* in P, find R(a), and compute $H_{p}\left(a\right)$ by Equation (5).11:    **end if**12:    Select the link $a_{h}$ which has the highest potential entropy in P, and identify its real label.13:    **if** the real label of $a_{h}$ is “negative” **then**14:        $a_{h}\leftarrow 0$, $A_{t}\leftarrow A_{t}\cup \left\{a_{h}\right\}$, $P\leftarrow P-\{a_{h}\}$15:    **else**16:        $a_{h}\leftarrow 1$17:        Find $R(a_{h})$ from P, for each link in $a_{r}$ in $R(a_{h})$, set $a_{r}\leftarrow 0$18:        $A_{t}\leftarrow A_{t}\cup \left\{a_{h}\right\}\cup R\left(a_{h}\right)$, $P\leftarrow P-\left\{a_{h}\right\}-R\left(a_{h}\right)$19:    **end if**20:    n←n+121:**end while**

#### 4.2.2. The *Biased Constrained Active Learning* Methods

As we discussed before, due to the sparsity of anchor links, acquiring enough informative positive anchor links under a limited cost is very important. However, in the *Normal Constrained Active Learning* methods, there may not be enough mechanisms to increase the probability of each identified link to be positive. So if we explore such a mechanism, and integrate it into the *Normal Constrained Active Learning* methods, we may achieve better results. Thus we present our *Biased Constrained Active Learning* methods, which prefer the potential positive links over the potential negative links in the query process.

According to the experiments in [1], in different circumstances, when predicting the existing anchor links, the *MNA* model can achieve higher accuracy than the *SVM* model contained in it by using the *one-to-one constraint*. So to our *Biased Constrained Active Learning* methods, the learner should firstly use the trained *MNA* model to predict the potential positive anchor links in P, and collect them in the set $A_{p}^{+}$. Then in each round of query, the link to be identified will be selected from $A_{p}^{+}$.

However, $A_{p}^{+}$ may also contain some links with low $P_{\theta }\left(1|a\right)$, since the current method doesn’t have a mechanism to filter out this kind of links. That is contrary to the goal of acquiring positive links. One way to solve this problem is to apply a threshold δ, and to any link $a\in A_{p}^{+}$, if $P_{\theta }\left(1|a\right)<\delta$, then delete *a* from $A_{p}^{+}$. However, since the *SVM* model θ changes after each time of query, the value of δ should also be updated with it. As a result, a novel entropy-based δ adjusting method is integrated in our *Biased Constrained Active Learning* methods. The main idea of it is: (1) Reducing the value of δ when the newly queried links have improved the effects of θ, so that in the next round of query, some links with smaller $P_{\theta }\left(1|a\right)$ but larger $H_{p}\left(a\right)$ can be identified; (2) Enlarging the value of δ when the newly queried links have not increased the effects of θ, so that to improve the probability of identifying positive links in the next round of query. At the beginning of each round of query, this δ adjusting method evaluates the effects of θ by computing $H_{new}^{P}$, which is the sum of all links’ entropies in P. If $H_{new}^{P}$ is smaller than the sum of these links’ entropies in the former round of query (which is represented by $H_{old}^{P}$), it means the uncertainty of θ to these links has decreased, i.e., the effects of θ have increased. In addition, in each round of query, the value of δ is updated by pulsing/deducting a number $\Delta_{\delta }\in \left(0,1\right)$. However, we can notice that if $H_{new}^{P}\geq H_{old}^{P}$ (i.e., the effects of θ haven’t been improved), the value of $\theta -\Delta_{\delta }$ can be smaller than $P_{old}^{+}$. Here $P_{old}^{+}$ is the equal to $P_{\theta }\left(1|a_{h}\right)$, which represents the probability of the identified link $a_{h}$ in the former round to be evaluated as positive by θ in the former round. In addition, since the effects of θ haven’t been improved in this round, the identified $a_{h}$ of the former round is likely to be a negative link which is wrongly predicted as “positive”. In addition, a good way to alleviate it is to make sure the value of $P_{\theta }\left(1|a_{h}\right)$ in this round is bigger than $P_{old}^{+}$, in this way to improve the probability of the identified link $a_{h}$ in this round to be “positive”. So if $H_{new}^{P}\geq H_{old}^{P}$, we can to set $\delta =Max\{P_{old}^{+},\delta +\Delta_{\delta }\}$ to improve the effects of the δ adjusting method.

After creating $A_{p}^{+}$, we should decide which potential positive anchor link to be identified from $A_{p}^{+}$. A simple way is to identify the link with the largest related link set, in this way to label as many links as possible in each round of query. However, by using this way, many of the identified positive links may contain little information with large related link sets, that is contrary to our goal of making sure the identified positive links containing enough information. As a result, here we firstly compute the potential entropy of each link in $A_{p}^{+}$, then identify the one with the highest potential entropy. Since in each round of query, the potential positive anchor link is identified from $A_{p}^{+}$, when δ is big enough, $A_{p}^{+}$ can be empty, and thus no link can be identified. So in order to ensure that a potential positive link can be identified from $A_{p}^{+}$ in each round of query, when $A_{p}^{+}$ is empty, we can use δ=δ−d to decrease the value of δ and then recreate $A_{p}^{+}$ until $A_{p}^{+}$ is not empty. Here d∈(0,1), and in our experiments we find that when setting d=0.1, the active learning methods can achieve good performances. So to simplify the process of parameter setting, we finally set d=0.1 in our algorithm. The proposed framework of the *Biased Constrained Active Learning* is shown in Algorithm 2. If we set $H_{p}=H_{B}$, then the algorithm is the *Biased Basic-entropy-based Constrained Active Learning (BBC)*; and if we set $H_{p}=H_{M}$, then the algorithm is the *Biased Mean-entropy-based Constrained Active Learning (BMC)*.

**Algorithm 2** The framework of *Biased Constrained Active Learning***Input:** Two heterogeneous social networks: $G^{s}$ and $G^{t}$. Two sets of labeled anchor link: The training set $A_{t}$ and the validation set $A_{v}$. The query pool P. The max number of queries $n_{q}$. The potential entropy computation method $H_{p}$. The threshold adjusting pace $\Delta_{\delta }$**Output:** The new training set $A_{t}$ and the new query pool P1:Initialize n←0, δ←0.5, $H_{old}^{P}\leftarrow +\infty$, $P_{old}^{+}\leftarrow 0.5$, d←0.12:For each *a* in $A_{t}$ and P, extract four types of features.3:**while** $n<n_{q}$ **do**4:    Train a *SVM* model θ on $A_{t}$ according to the training part in *MNA*.5:    For each *a* in P, use θ to predict $P_{\theta }\left(0|a\right)$ and $P_{\theta }\left(1|a\right)$.6:    $H_{new}^{P}\leftarrow \sum_{a}^{P}H_{B}\left(a\right)$7:    **if** $H_{new}^{P}<H_{old}^{P}$ and δ>0 **then** $\delta \leftarrow \delta -\Delta_{\delta }$8:    **else if** $H_{new}^{P}\geq H_{old}^{P}$ **then** $\delta \leftarrow Max\{P_{old}^{+},\delta +\Delta_{\delta }\}$9:    **end if**10:    Use θ as the trained classifier in *MNA*, and use *MNA* to predict the labels of all links in P, and collect all of the predicted positive links into the set $A_{p}^{+}$. Then set ${\hat{A}}_{p}^{+}\leftarrow \left\{a|a\in A_{p}^{+},P_{\theta }\left(1|a\right)\geq \delta \right\}$.11:    **while** ${\hat{A}}_{p}^{+}$ is ∅ **do**12:        δ←δ−d, ${\hat{A}}_{p}^{+}\leftarrow \left\{a|a\in A_{p}^{+},P_{\theta }\left(1|a\right)\geq \delta \right\}$13:        **if** δ<0 **then** ${\hat{A}}_{p}^{+}\leftarrow P$14:        **end if**15:    **end while**16:    $A_{p}^{+}\leftarrow {\hat{A}}_{p}^{+}$17:    **if** $H_{p}$ is $H_{B}$ **then**18:        For each *a* in $A_{p}^{+}$, compute $H_{p}\left(a\right)$ by Equation (2)19:    **else**20:        Use *MNA* to predict the labels of all links in $A_{v}$, and compute $P_{YY}$ and $P_{NY}$ by Equation (4)21:        For each *a* in $A_{p}^{+}$, find R(a) and compute $H_{p}\left(a\right)$ by Equation (5)22:    **end if**23:    Select the link $a_{h}$ which has the highest $H_{p}\left(a\right)$ in $A_{p}^{+}$, and identify its real label, and set $P_{old}^{+}\leftarrow P_{\theta }\left(1|a_{h}\right)$24:    **if** the real label of $a_{h}$ is “negative” **then**25:        $a_{h}\leftarrow 0$, $A_{t}\leftarrow A_{t}\cup \left\{a_{h}\right\}$, $P\leftarrow P-\{a_{h}\}$26:        $H_{old}^{P}\leftarrow H_{new}^{P}-H_{B}\left(a_{h}\right)$27:    **else**28:        $a_{h}\leftarrow 1$29:        Find $R(a_{h})$ from P. For each $a_{r}$ in $R(a_{h})$, set $a_{r}\leftarrow 0$30:        $A_{t}\leftarrow A_{t}\cup \left\{a_{h}\right\}\cup R\left(a_{h}\right)$ ,$P\leftarrow P-\left\{a_{h}\right\}-R\left(a_{h}\right)$31:        $H_{old}^{P}\leftarrow H_{new}^{P}-H_{B}\left(a_{h}\right)-\sum_{a}^{R(a_{h})}H_{B}\left(a\right)$32:    **end if**33:    n←n+134:**end while**


## 5. Experimental Section

### 5.1. Data Preparation

In this paper, we collect two datasets according to the way in [1]. One is from Foursquare, a popular location-based online social network, and the other is from Twitter, one of the hottest microblogging social networks. A more detailed comparison of these two datasets is available in Table 1. In order to conduct experiments, we pre-process these raw data to obtain the ground-truth of users’ anchor links. In Foursquare network, we can collect some users’ Twitter IDs in their account pages. We use these information to build the ground-truth of anchor links between user accounts across the two networks. If a Foursquare user has shown his/her Twitter ID in the website, we treat it as an anchor link between this user’s Foursquare account and Twitter account. In this way, we obtain 600 anchor links. For more information about the datasets and the crawling method, please refer to [1].

### 5.2. Experiment Setups

In order to evaluate the effectivenesses of these compared methods on anchor link prediction, we select three different metrics in terms of F1-measure (F1), Precision (Prec.), Recall (Rec.).

We design several groups of experiments and use all the 600 anchor links as the set of positive anchor links. Before each experiment, according to the predefined *Negative-Positive Rate* ($R_{NP}$, $R_{NP}=\frac{\#negative\_pairs}{\#positive\_pairs}$), we randomly sampled a set of non-existing anchor links between these 2 user sets as the negative anchor link set. These links are partitioned into 4 parts with 6 folds cross validation: 1 fold as the general training set, 2 folds as the query set, 1 fold as the validation set and the remaining 2 folds as the test set.

In each group of experiments, we randomly select links of our training set $A_{t}$ from the general training set. The parameter used to control the percentage of selected links in the general training set is $R_{t}$. In addition, the threshold adjusting pace for *BBC* and *BMC* is set as $\Delta_{\delta }=0.01$. The query set is used as the query pool P, the validation set $A_{v}$ is used to calculate $P_{YY}$ and $P_{NY}$ for *MC, BMC*.

### 5.3. Effectiveness Experiments

It is known that our proposed constrained active learning methods are integrated with the *MNA* method, in this way to improve its performances on anchor link prediction. So in this subsection, we will conduct two groups of experiments to analyze the effectiveness of our methods on improving the performances of *MNA*.

Here we select four sampling methods as the baseline methods, so in total, there are eight methods to be compared. The compared methods are summarized as follows:

- *The Normal Constrained Active Learning methods*: The first kind of proposed methods in this paper, including the *Basic-entropy-based Constrained Active Learning (BC)* and *Mean-entropy-based Constrained Active Learning (MC)*.
- *The Biased Constrained Active Learning methods*: The second kind of proposed methods in this paper, including the *Biased Basic-entropy-based Constrained Active Learning (BBC)* and *Biased Mean-entropy-based Constrained Active Learning (BMC)*.
- *MNA*: A state-of-art supervised anchor link prediction method based on heterogeneous features and outperforms [1], which doesn’t do any query to enlarge its original training set.
- *The Entropy-based Active Learning (EA)*: A method widely used in some state-of-art link query problems, such as data integration [41], semi-supervised clustering of links [42].
- *The Random Query Methods*: Two base-line query methods integrated to *MNA* for better comparison. One is the *Normal Random Query (NRQ)* which only adds the randomly queried links to $A_{t}$; the other is the *Constrained Random Query (CRQ)*, which adds not only the randomly queried links, but also the related link set of each queried positive link to $A_{t}$.

For fair comparisons, each of these compared methods uses the same parameter set to train the *MNA* model and predicts anchor links by the trained model.

**Figure 2:**
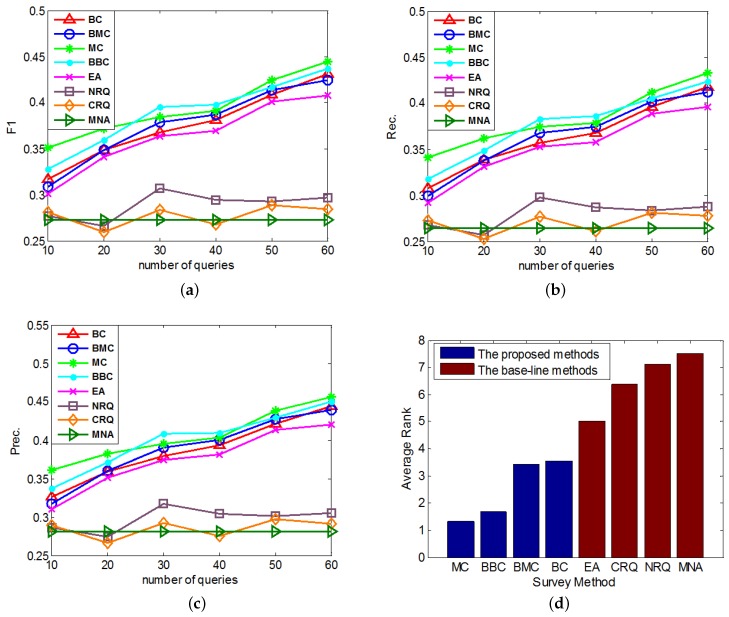
The comparisons of different query methods’ performances on anchor link prediction. We do different numbers of queries in the query set. (**a**) F1-measure; (**b**) Precision; (**c**) Recall; (**d**) Average Rank.

In the first group of experiments, we study the performances of the proposed active learning methods on anchor link prediction with different numbers of queries when the degree of data imbalance is given. In addition, in real-world anchor link prediction, the negative anchor links are much more than the positive anchor links, so in this group of experiments we can set $R_{NP}$ to be a number which is obviously larger than 1.0. We have done several tests and find that when setting $R_{NP}$ to different values which are obviously larger than 1.0 (e.g., 5, 10, 15, 20, 40), similar conclusions can be drew from the performance comparisons. So here we set $R_{NP}=10.0$, and conduct the performance comparisons. In each round of the cross validation, we set $R_{t}=0.5$ and use different methods to query 0,10,20,⋯,60 times in the query pool P, then add the acquired links to the training set $A_{t}$. The performances of all compared methods under different number of queries are compared in Figure 2a–c. And each method’s average performance rank, which is averaged over its performance ranks on different numbers of queries in all of these 3 figures (e.g., in each of these 3 figures, *MC*’s list of performance ranks on different numbers of queries is {1,1,2,2,1,1}, so its average rank over these 3 figures is (4×1+2×2)×3/(6×3).), is shown in Figure 2d.

**Figure 3:**
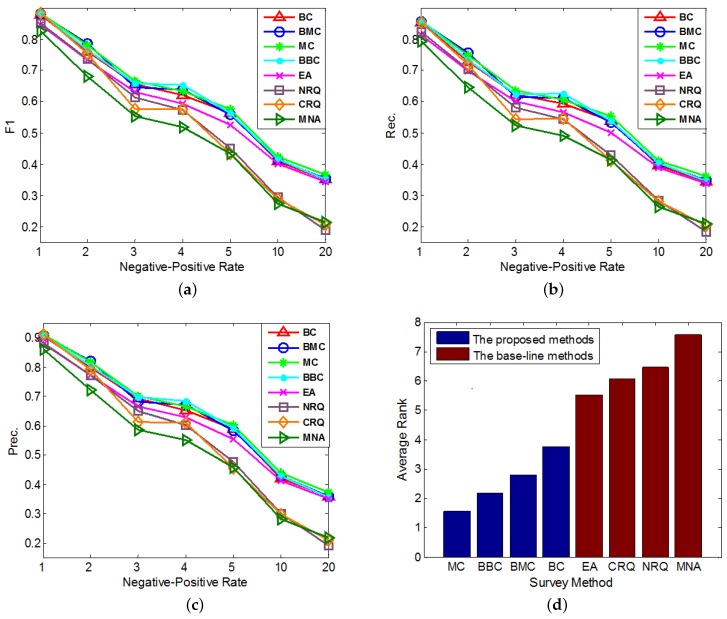
The comparisons of different query methods’ performances on anchor link prediction. We use different *Negative-Positive Rates* in both training and test sets. (**a**) F1-measure; (**b**) Precision; (**c**) Recall; (**d**) Average Rank.

In the second group of experiments, we set $R_{t}=0.5$ and test the performances of our methods with different imbalanced datasets. In each round of the cross validation, we sample anchor links as the experimental data samples according to different imbalance ratios, i.e., *Negative-Positive Rates* ($R_{NP}$). In addition, in each round of experiment, we set the number of queries for each method (except *MNA*) as 60. The performances of all compared methods under different imbalance ratios are compared in Figure 3a–c. In addition, similar to Figure 2d, each method’s average performance rank, which is averaged over its performance ranks on different *Negative-Positive Rates* in all of these 3 figures, is shown in Figure 3d.

According to the results in Figure 2 and Figure 3, we can analyze and conclude as follows:

- Overall, *MC* outperforms other methods on the anchor link prediction. However, the listed performances of *BBC* are not much worse than *MC*. In addition, compared with *BBC*, *MC* needs a reasonable validation set to do anchor link prediction. So to the anchor link prediction problem, *MC* would be the best choice if we have enough labeled anchor links to form a reasonable validation set. However, when the labeled anchor links in the validation set are not sufficient, the sample distribution of the validation set can be very different from the sample distribution of the overall experimental data. In addition, thus according to what we analyzed in the definition of Equation (5), the computed potential entropies of *MC* in the query pool are not precise, which can result in the bad performances of *MC*. So in this circumstance, *BBC* would be a better choice.
- According to the average rank of each compared method, *BBC* performs better than *BC*. However, *BMC* cannot perform better than *MC*. To understand this, we can suppose that there exist two anchor links in P, whose basic entropies are the same, and related link sets contain the same amount of information. In addition, thus from these two links, the *MC* prefers to identify the one with bigger $P_{M}\left(1|a\right)$ (See Equation (5)). Furthermore, according to the definition of $P_{M}\left(1|a\right)$, the bigger $P_{M}\left(1|a\right)$ is, the more likely *a* is to be a positive anchor link. So we can see *MC* already has a reasonable mechanism to prefer the potentially positive links. As a result, adding a new mechanism of preferring potential positive links is not easy to make *MC* perform better. Because this new mechanism may make *MC* focus on identifying the links that are easy to be correctly predicted as positive, but neglect some informative links whose labels are hard to be correctly predicted. And this may also be the reason why *BMC* cannot outperform *BBC* in these experiments.
- All of our proposed *Constrained Active Learning* methods perform better than *EA*, it proves the value of applying the one-to-one constraint to the active learning in anchor link prediction problem.
- In the same experiment, the performance ranks of each method on metrics F1, Prec. and Rec. are almost the same.

### 5.4. Portability Experiments

The proposed constrained active learning methods are integrated with the *MNA* method. And among them, we have proved that the *MC* and *BBC* methods have great values on improving the performances of *MNA* in the previous experiments. However, whether our constrained active learning methods can also work well on other existing anchor link prediction techniques remains to be proved. So in this subsection, we will firstly change the basic anchor link prediction method of the proposed approach (Here, we use *M* to represent it) in different ways: (1) set $M=M_{1}$ when the classifier of *M* is replaced with the one in [10]; (2) set $M=M_{2}$ when the classifier of *M* is replaced with the one in [9]; (3) set $M=M_{3}$ when the *One-to-One matching algorithm* of *M* is changed to the *GUARD* algorithm, which is proposed in [30]. Then we will conduct experiments to test the effects of *MC* and *BBC* together with other baseline query methods on improving the performances of $M_{1}$, $M_{2}$ and $M_{3}$, respectively. In this way to analyze their portability to other state-of-art supervised anchor link prediction techniques.

Similar to Section 5.3, the compared methods in each group of the portability experiments are as follows:

- *M*: The basic anchor link prediction method (which can be set as $M_{1}$, $M_{2}$ or $M_{3}$). It will be directly used as a base-line method.
- *MC-M*: Integrating the proposed *Mean-entropy-based Cons- trained Active Learning (MC)* method to *M*.
- *BBC-M*: Integrating the proposed *Biased Basic-entropy-based Constrained Active Learning (BBC)* method to *M*.
- *EA-M*: Integrating the *Entropy-based Active Learning (EA)* method to *M*. It will be used as a base-line method.
- *NRQ-M and CRQ-M*: Two base-line query methods integrated to method *M* for better comparison. One is the *Normal Random Query (NRQ)*; the other is the *Constrained Random Query (CRQ)*.

**Figure 4:**
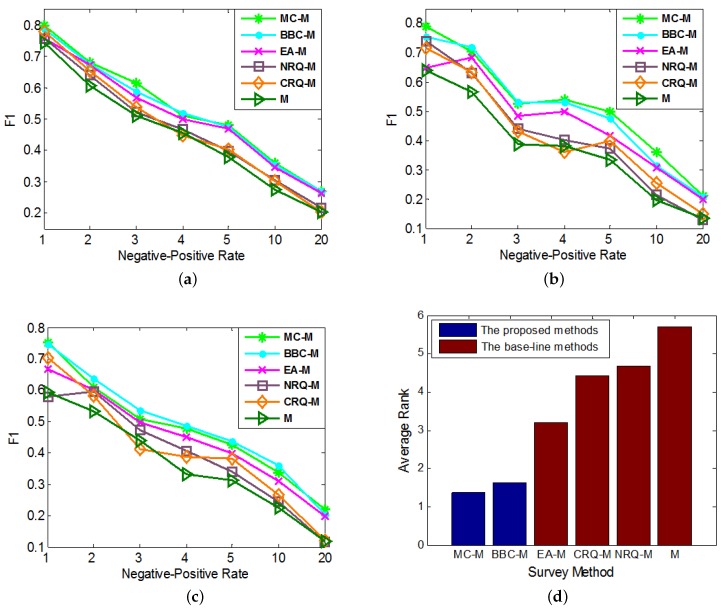
The comparisons of different query methods’ effects on improving the performances of different anchor link prediction techniques. We use different *Negative-Positive Rates* in both training and test sets. (**a**) $M=M_{1}$; (**b**) $M=M_{2}$; (**c**) $M=M_{3}$; (**d**) Average Rank.

In the experiments, we test the performances of these compared methods with different imbalanced datasets. In each round of cross validation, we sample anchor links as the experimental data samples according to different imbalance ratios, i.e., Negative-Positive Rates ($R_{NP}$). In addition, in each round of experiment, we set $R_{t}=0.3$ and the number of queries for each method (except *M*) as 30. From the results, we notice that the performance ranks of each method on metrics F1, Prec. and Rec. in each experiment are almost the same, that is consistent with what we concluded from the experimental results in SubSection 5.3. As a result, in order to save the space, in Figure 4, we only show the performance comparison results on metric F1.

As it shows in Figure 4, the effects of *MC* and *BBC* together with other baseline query methods on improving the performances of $M_{1}$, $M_{2}$ and $M_{3}$ are compared in Figure 4a–c, respectively. In addition, similar to Figure 2d, each query method’s average performance rank, which is averaged over its performance ranks on different *Negative-Positive Rates* in all of these 3 figures, is shown in Figure 4d. From the results, we can see our *MC* and *BBC* methods perform significantly better than other base-line query methods on improving the performances of other state-of-art supervised anchor link prediction techniques. In addition, in most cases, *MC* can achieve better effects than *BBC*. As a result, we can convince that our *MC* and *BBC* have great values on improving the performances of many different supervised anchor link prediction techniques, and it is better to choose *MC* when enough labeled links can be collected to form a reasonable validation set.

## 6. Conclusions

This paper is the first to describe and study the problem of applying active learning on anchor link prediction across multiple heterogeneous social networks. Based on the *one-to-one constraint* of the anchor link prediction problem, we design several *Constrained Active Learning* methods. Different from the traditional query methods, our constraint active learning methods can label more than one link after an unlabeled anchor link has been queried. Furthermore, we design different mechanisms, so that to make sure our methods can acquire more information when the maximum number of queries and sample set for query have been given. We choose the *MNA* method as our basic anchor link prediction method, and conduct our experiments on the anchor links between two real-world social networks, Foursquare and Twitter. Then we conduct experiments to test the effects of our *MC* and *BBC* methods on improving the performances of other state-of-art anchor link prediction techniques. The results show that our *MC* and *BBC* can adapt to many different supervised anchor link prediction models, and in general, *MC* outperforms other compared query methods on anchor link prediction. However, the *BBC* method can be a better choice if we do not have enough labeled anchor links to form a reasonable validation set but want to achieve good enough results. Our future works include the study on how to select a validation set with a moderate size for the *MC* method, and the computation for the optimal value of the parameter $\Delta_{\delta }$ in Algorithm 2.

## Figures and Tables

**Table 1:** Properties of the Heterogeneous Social Networks.

	Property	Network	
		Twitter	Foursquare
# node	user	600	600
	user/tip	889,925	9012
	location	41,196	7578
# link	friend/follow	6640	3611
	write	889,925	9012
	locate	48,268	9012
